# *Snail-1* Overexpression Correlates with Metastatic Phenotype in BRAF^V600E^ Positive Papillary Thyroid Carcinoma

**DOI:** 10.3390/jcm9092701

**Published:** 2020-08-21

**Authors:** Katarzyna Wieczorek-Szukala, Janusz Kopczynski, Aldona Kowalska, Andrzej Lewinski

**Affiliations:** 1 Department of Endocrinology and Metabolic Diseases, Medical University of Lodz, 93-338 Lodz, Poland; katarzyna.wieczorek@umed.lodz.pl; 2 Department of Pathology, Holy Cross Cancer Center, 25-734 Kielce, Poland; janusz.kopczynski@onkol.kielce.pl; 3 Endocrinology Clinic, Holy Cross Cancer Center, 25-734 Kielce, Poland; aldona.kowalska@onkol.kielce.pl; 4 Faculty of Medicine and Health Sciences, Jan Kochanowski University, 25-319 Kielce, Poland

**Keywords:** PTC, thyroid, metastasis, Snail-1, BRAF^V600E^

## Abstract

The ability of cancer to metastasize is regulated by various signaling pathways, including transforming growth factor β (TGFβ), also implicated in the upregulation of Snail-1 transcription factor in malignant neoplasms. B-type Raf kinase gene (BRAF)^V600E^, the most common driving mutation in papillary thyroid carcinoma (PTC), induces epithelial to mesenchymal transition (EMT) in thyroid cancer cells through changes in the Snail-1 level, increasing cell migration and invasion. However, little is known about the mechanism of Snail-1 and BRAF^V600E^ relations in humans. Our study included 61 PTC patients with evaluated BRAF^V600E^ mutation status. A total of 18 of those patients had lymph node metastases—of whom 10 were BRAF^V600E^ positive, and 8 negative. Our findings indicate that the expression of Snail-1, but not TGFβ1, correlates with the metastatic phenotype in PTC. This is the first piece of evidence that the upregulation of Snail-1 corresponds with the presence of BRAF^V600E^ mutation and increased expression of Snail-1 in metastatic PTC samples is dependent on BRAF^V600E^ mutation status.

## 1. Introduction

Papillary thyroid carcinoma (PTC) is the most common thyroid cancer accounting for 80–85% of all thyroid cancer cases. Although its incidence is gradually growing, the cancer-related mortality rate is relatively stable, and PTC has a good prognosis with an average 5-year survival rate of above 90% [1]. However, above 50% of PTC case often have performed local lymph node metastasis and approximately 10% of PTC cases represent aggressive metastatic neoplasm with distant metastases to lungs or bones [2]. In addition to an increased rate of primary tumors, there has been also growing incidence of regional and distant PTC metastases, which obviously increases the global need for improving our understanding of pathogenesis process and identifying effective, targeted treatments.

The fundamental mechanism by which epithelial-derived tumor cells may become malignant and obtain an invasive phenotype is the epithelial to mesenchymal transition (EMT). EMT is essential to both physiological developmental processes, as well as metastatic cancer. Numerous studies have demonstrated that this process is aberrantly activated during thyroid cancer development [3,4]. Moreover, it was discovered both in vitro and in vivo that thyroid tumour cells from PTCs and anaplastic cancer (ATCs) may constitutively display an active EMT process when compared to normal thyrocytes. This results in the loss of cells’ polarity, decreased expression of particular epithelial markers and increased expression of mesenchymal markers [5,6].

It is currently known that EMT may be initialized and maintained by various molecular factors, including an inflammatory cytokine-transformation growth factor-β (TGF-β) [3]. Transforming growth factor β (TGFβ) is one of the most abundant cytokines that regulates many biological processes such as cell differentiation, apoptosis, proliferation, and other physiological and pathological conditions. TGFβ1 is thought to be the primary isoform which is overexpressed in many tumor types and may promote tumor invasion and metastasis through multiple Smad-independent or non-canonical signaling pathways [7,8]. The TGFβ family of signaling molecules has been implicated in the upregulation of key transcription factors such as Twist, Zeb or Snail-1 [8,9]. Importantly, it has been also discovered that such transcription factors may initiate the EMT process itself [3,10].

Snail-1 belongs to a family of zinc-finger transcription factors and its primary function is the repression of E-cadherin, which results in reduced cell adhesion and promotes migratory capacity [9,11]. In addition, Snail-1 activation induces fibrosis in various organs such as the kidneys, liver and lungs. The pathological activation of Snail-1 probably contributes to organ fibrosis which may further lead to chronic inflammation and cancer [12]. Snail-1 has been also recently implicated in the regulation of drug resistance and the induction of the cancer stem cell (CSC) phenotype [9,13].

However, the induction of the EMT process in the context of cancer invasion remains Snail-1′s most studied function. A large number of studies have found that metastatic tumors of the colon, breast, ovary and prostate carcinomas overexpress the Snail-1 protein [9,14]. Its overexpression is generally correlated with aggressiveness, lymph node metastases (LNM), tumor recurrence and poor prognosis [15,16]. Our previous experiments concerning colon adenocarcinoma HT29 cells also indicated that Snail-1 overexpression reduced cell adhesion and increased migratory properties, which coincides with the above observations [17]. Furthermore, it was shown that Snail-1 may also play a key role in cancer cell cytoskeleton reorganization, while a positive correlation between Snail-1 presence and Tubulin–β3, (TUBB3) (one of the main proteins building microtubules, a major component of the eukaryotic cytoskeleton) upregulation in adenocarcinoma cell lines has been observed [18]. Although Snail-1 is not expressed in normal thyroid tissue, it was described to be overexpressed in different thyroid cancer cell lines [19].

PTC oncogenesis is associated with the occurrence of the mutations within the B-type Raf kinase gene–BRAF [20]. Although numerous different variants of BRAF mutations have been discovered so far, over 90% are the BRAF^V600E^ mutation. This genetic aberration influences and constantly activates the mitogen-activated protein kinase (MAPK) signaling pathway and leads to uncontrolled cell proliferation and differentiation [21]. Among other genetic alternations involving the MAPK pathway (such as RET/PTC or RAS) BRAF^V600E^ is present in more than 50% of PTC, but is rare in follicular variants, and not found in follicular thyroid cancer. Therefore, BRAF^V600E^ has been postulated as a good prognostic marker to assist in risk stratification for patients with PTC [22].

The tumorigenic role of BRAF^V600E^ in PTC development was well documented before in BRAF^V600E^ transgenic mice models. Studies performed on rat thyroid cells overexpressing BRAF^V600E^ suggested that BRAF^V600E^ is an initiator of tumorigenesis and is required for tumor progression in PTC [23]. A large number of clinical studies have demonstrated an association of BRAF^V600E^ mutation with aggressive clinicopathologic characteristics and high tumor recurrence, although the results are controversial [24].What is important, it has also been shown that BRAF^V600E^ plays a crucial role in direct EMT induction. Baquero et al. have studied this phenomenon in thyroid cancer cells and reported that BRAF^V600E^ induces EMT through changes in Snail-1 and E-cadherin protein expression levels, which in turn, increase cell migration and invasion [25]. The interaction of BRAF^V600E^ and Snail-1 has been observed also in other cancer types. Interestingly, Massoumi et al. have reported that hyperactivation of the BRAF^V600E^mutant in melanoma cells resulted in Snail-1 overexpression and increased their metastatic potential. Introducing mutated BRAF into primary melanocytes led to increased Snail-1 expression after malignant transformation, constitutively high ERK activity and resulted in acquisition of a more aggressive cell phenotype [26].

An association has been suggested between BRAF^V600E^ and TGFβ in aggressiveness induction of PTCs. It has been shown in rat thyroid cells overexpressing BRAF^V600E^ that this oncogene stimulates TGFβ secretion, and both proteins exert the same effect on both E-cadherin expression and cell invasion [27]. However, the above studies, based on animal or cell line models (mostly anaplastic thyroid cancer model), may not fully represent the interactions between Snail-1 and BRAF^V600E^ mutation in PTC in vivo.

The aim of our study was to determine whether there is any correlation between *Snail-1* and *TGFβ1* gene expression and the existence of BRAF^V600E^ mutation in patients with metastatic or non-metastatic PTC. To our knowledge, this study is the first attempt assessing the expression of the above EMT markers and the metastatic potential of PTC in paraffin slides containing human PTC collected during surgery.

## 2. Materials and Methods

### 2.1. Patients

**Figure 1:**
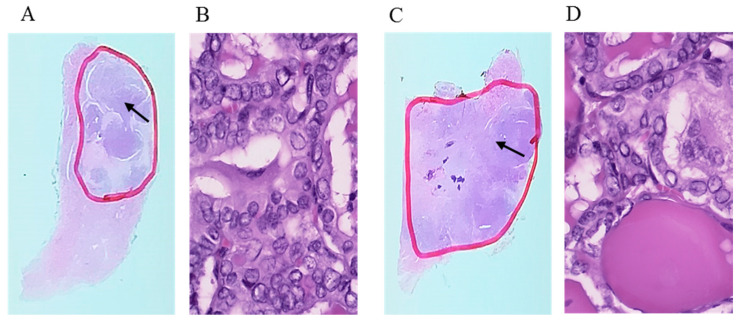
Representative cancer foci images. Hematoxylin and eosin staining of 2 different papillary thyroid carcinoma (PTC) samples-the primary tumour foci have been marked by pathologist (red line) (**A**,**C**). The images (**B**,**D**) show magnification ×400.

The study group was recruited from PTC patients diagnosed and recruited to the trial during routine follow-up from 2012 at the Endocrinology Clinic of the Holy Cross Cancer Center (Kielce, Poland). Thin-section paraffin-embedded slides of thyroid tissues were prepared by a Holycross Cancer Centre pathologist at the time of diagnosis as decribed earlier [28,29]. One of our pathologists (J. K.) marked the area containing primary tumour foci on one haematoxylin and eosin-stained slide (red line, Figure 1).

A total of 61 PTC specimens were chosen for further analysis. All the study procedures were approved by the Bioethical Committee at the Holy Cross Medical Chamber, Kielce (no. 2/2013). Patients provided their written consent for molecular tests.The patient characteristics are described in Table 1.

The presence of the V600E mutation in the BRAF gene was evaluated by three genotyping methods: allele-specific amplification PCR (ASA-PCR), qPCR, and Seq as described previously by Kowalik et al. [29]. Within the 61 patients in this study, 30 samples were BRAF^V600E^ positive and 31 BRAF^V600E^ negative. Within 18 patients with lymph node metastasis–10 were BRAF^V600E^ positive, and 8 negative. Controls were obtained from surrounding normal tissue after pathologist supervision.

### 2.2. Molecular Methods

The pathologist marked the area containing PTC tumor cells on a hematoxylin and eosin-stained slide. Then, 4 to 5 unstained slides were deparaffinized by soaking twice in xylene for 15 min, twice in 96% alcohol for 5 min and once in distilled water. Tissue was scraped from thepathologist-selected area with a scalpel and transferred to a test tube for further RNA isolation.

RNA isolation has been performed using a High Pure FFPE RNA Isolation Kit (Roche, Switzerland), according to the manufacturer’s protocol. The purity and integrity of total RNA was assessed by an Agilent 2100 Bioanalyzer. The degradation rate of total RNA was determined using RIN values. Only the samples with RIN > 7 were further analyzed. Total RNA was used in the first strand cDNA synthesis with a Maxima First Strand cDNA Synthesis Kit for RT-qPCR with dsDNase (Thermo Scientific, Waltham, MA, USA) according to manufacturer’s instructions. RT-qPCR was performed on the ABI PRISM^®^ 7500 Sequence Detection System (Applied Biosystem, CA, USA) by using TaqMan Gene Expression Assays and TaqMan™ Fast Advanced Master Mix according to the manufacturer’s specification. The Assays Identification numbers were: SNAI1-Hs00195591_m1; TGFβ1-Hs00998133_m1; GAPDH Hs99999905_m1. Thermal cycler conditions were as follows: 2 min at 50 °C, hold for 20 s at 95 °C, followed by two-step PCR for 40 cycles of 95 °C for 3 s followed by 60 °C for 30 s. Amplification reactions, in triplicate for each sample, were performed and the results were normalized to the GAPDH gene expression level. An analysis of relative gene expression data was performed, using the 2–ΔΔCT method. The calibrator was prepared as mean gene expression values for normal tissue from 12 patients.

### 2.3. Statistics

The statistical analysis was carried out using the Statistica 12 software (StatsoftPolska, Kraków, Poland). A graphical representation of the results was prepared using the SigmaPlot 11 software (Systat Software Inc., San Jose, CA, USA). The results are presented as the mean of three independent experiments ± SD. The normality of the distribution was assessed using the Shapiro–Wilk test. The Student’s *t*-test was used for normally distributed parameters. Chi-square tests and *t*-test were used in the medical history analysis. In all the analyses, results were considered statistically significant when *p* < 0.05.

## 3. Results

### 3.1. Snail-1, but not TGFβ1 Expression is Correlated with Metastatic Phenotype in PTC

**Figure 2:**
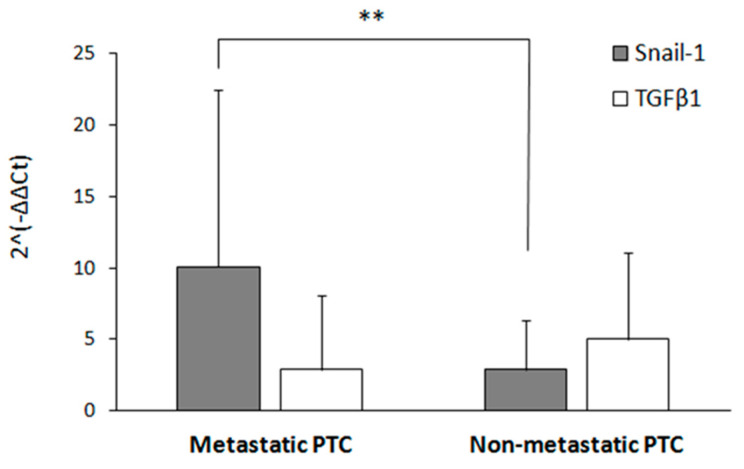
The expression of *Snail-1* but not *TGFβ1* was correlated with the metastatic phenotype in PTC samples. The gene expression was evaluated by quantitative RT-qPCR in metastatic (*n* = 18) and non-metastatic (*n* = 43) PTC groups. The relative mRNA level was normalized to GAPDH. The results are presented as the mean of three independent experiments ± SD. ** *p* < 0.001.

The EMT process may be induced both through transcription factors such as Snail-1 or growth factor TGFβ1. Altered expression of those genes has been observed previously in thyroid cancer cell line models [4,25]. We analyzed the expression of those two genes in 61 FFPE tissues from PTC patients. A total of 18 PTC samples were representing metastases (18 lymph node, with three distant—as described in Table 1)—described later as “Metastatic PTC”, and 43 PTC samples had no diagnosed metastases (“Non-metastatic PTC”). RT-qPCR was performed using TaqMan Gene Expression Assays. We observed a statistically significant (*p* < 0.001), more than 2.5-fold increase in *Snail-1* gene expression in metastatic PTC samples as compared to non-metastatic samples (Figure 2, grey bars). The expression of the *TGFβ1* gene in non-metastatic PTC patients was slightly higher, however, no statistical significance for this tendency between the studied groups was noted.

### 3.2. The Presence of BRAF^V600E^ Mutation Associates with Snail-1 Expression in PTC

**Figure 3:**
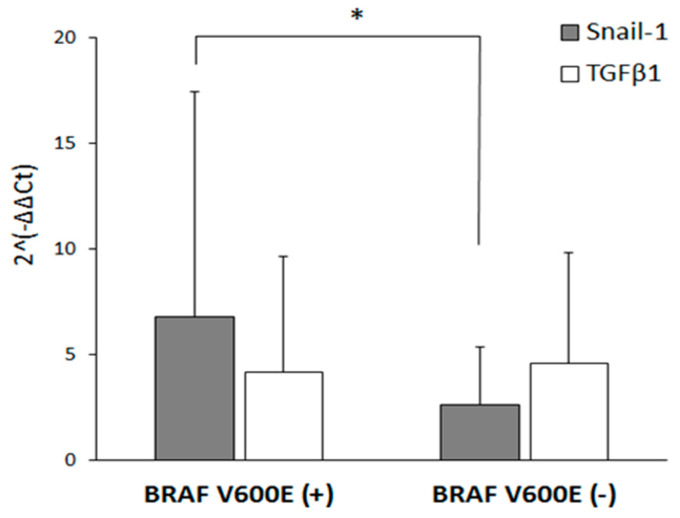
The expression of *Snail-1* is correlated with the presence of B-type Raf kinase gene (BRAF)^V600E^ mutation in PTC samples. The gene expression was evaluated by quantitative RT-qPCR in BRAFV600E (+) (*n* = 30) and BRAFV600E (−) (*n* = 31) PTC groups. The relative mRNA level was normalized to GAPDH. The results are presented as the mean of three independent experiments ± SD. * *p* < 0.05.

As described before, Snail-1 protein expression may be regulated by BRAF^V600E^ mutation in human thyroid cancer cells [25]. Therefore, we divided our studied group into BRAF^V600E^ positive (30 patients) and BRAF^V600E^ negative samples (31 patients). The presence of the V600E mutation in BRAF gene was evaluated previously [14]. *Snail-1* mRNA expression was significantly higher (ca. 2-fold) in PTC with BRAF^V600E^ mutation in comparison to BRAF^V600E^ negative samples (*p* < 0.05) (Figure 3). However, the expression *TGFβ1* did not differ between those two groups.

### 3.3. The Upregulation of Snail-1 in Metastatic PTC Samples is Dependent on BRAF^V600E^ Mutation Status

Furthermore, we aimed to evaluate the role of BRAF^V600E^ mutation on the expression of *Snail-1* and *TGFβ1* within metastatic PTC samples. Among 18 PTC patients with lymph node metastasis–10 samples harboured BRAF^V600E^ mutation (described later as “Metastatic- BRAF V600E (+)”), and 8 were BRAFV600E negative (“Metastatic–BRAF V600E (−)”). Among 43 PTC patients with no diagnosed metastases—20 were BRAF^V600E^ positive (“Non-metastatic–BRAF V600E (+)”), and 23 BRAF^V600^E negative (“Non-metastatic–BRAF V600E (−)”).

**Figure 4:**
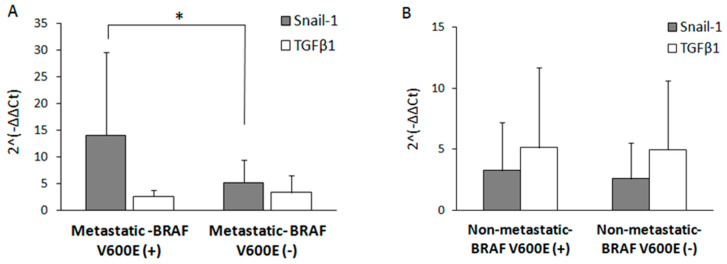
The upregulation of *Snail-1* in metastatic PTC samples is dependent on BRAF^V600E^ mutation status. The gene expression was evaluated by quantitative RT-qPCR in both BRAF V600E (+) (*n* = 10) and BRAF V600E (−) metastatic (*n* = 8) (**A**) and non-metastatic BRAF V600E (+) (*n* = 20) and BRAF V600E (−) (*n* = 23) PTC groups (**B**). The relative mRNA level was normalized to GAPDH. The results are presented as the mean of three independent experiments ± SD. * *p* < 0.05.

*Snail-1* expression level was nearly 3-fold higher in metastatic PTC that harbored the BRAF^V600E^ mutation when compared to the metastatic but BRAF^V600E^ negative group (*p* < 0.05) (Figure 4A, grey bars). Interestingly, the expression level of the *Snail-1* gene in metastatic but BRAF^V600E^ negative PTC samples (Figure 4A) remained on a similar level as in the non-metastatic PTC patients, regardless of BRAF^V600E^ mutation status (Figure 4B).

On the other hand, in both metastatic and non-metastatic PTC groups: BRAF V600E (+) or (−) (Figure 4A,B, white bars), no significant differences in *TGFβ1* gene expression levels were found (Student’s *t*-test, *p* > 0.05 in each case).

Table 2 summarizes some vital clinical features of the studied metastatic and non-metastatic PTC patient groups. Statistical analysis showed no significant statistical correlation between BRAF^V600E^ mutation status in PTC tissue and gender, age at diagnosis, or T-classification of the primary tumor. However, it was observed that significantly more samples of non-metastatic PTC tumors with T3–T4 classification harbored the BRAF^V600E^ mutation. Furthermore, it can be observed that more patients with non-metastatic and BRAF^V600E^ negative PTC had been diagnosed as less advanced T1–T2 tumor stage (*p* = 0.0002). Nevertheless, due to the limited number of patients in the studied groups evaluation of such analysis requires further research.

## 4. Discussion

PTC is the most common of all thyroid cancers, with a gradually growing incidence. The majority of PTC cases generally present good prognosis with conventional therapies. Nevertheless, approximately 10% of PTC cases progress to more aggressive forms associated with local invasion, distant metastases and poorer clinical outcome [1,2]. Despite metastasis remaining the key cause of failure in cancer treatment and mortality, its molecular mechanisms remain poorly examined.

Metastatic process is associated with change of phenotype due to the epithelial–mesenchymal transition (EMT), an essential physiological mechanism in embryonic development and tissue repair. However, EMT may also contribute to the progression of disease, such as organ fibrosis or cancer [3]. This process results from the induction of transcription factors that change gene expression, therefore promoting the loss of cell–cell adhesion, modifying cytoskeletal dynamics and leading to a change from epithelial morphology and physiology to the mesenchymal phenotype. EMT can be induced by various signaling pathways, for example mediated by transforming growth factor β (TGFβ), Wnt–β-catenin, Notch, Hedgehog receptor tyrosine kinases (RTK) [4,5].

The role of TGFβ in cancer is complex: this pathway may function both in tumor suppression and in tumor promotion. TGFβ also functions as a tumor suppressor in early tumor development. In a number of human cancers, TGFβ inhibits cell cycle progression, increases apoptosis, and suppresses the expression of growth factors, cytokines, and chemokines. The function of *TGFβ* as a tumor suppressor or a tumor promoter depends on the context and stage of tumor progression. Its shift to a pro-metastatic role at later tumor stages has been shown to be mediated by various signaling pathways [7,10].

The thyroid gland expresses the *TGFβ1* gene mRNA and synthesizes the protein, which under physiologic conditions regulates thyroid growth and function. Interestingly, it was concluded that the alterations in the signaling pathway of *TGFβ1* are not the same in tumors from different species [7]. It seems it is not entirely possible to apply the results obtained in animal or cell culture studies to normal or pathological human thyroid tissue. It should be noted that using a single experimental research model usually entails some limitations. For example, employing exclusively tissue material collected from patients, may lead to a somehow less comprehensive scientific reasoning.

The studies of the expression of *TGFβ1* performed before in human PTC revealed that, when compared to the central areas, the peripheral invasive areas of the tumors had increased expression of *TGFβ1*, *NFkβ*, and CDC42 as well as showing a decrease in cell–cell adhesion proteins. Moreover, these regions presented also an overexpression of vimentin, a characteristic marker of EMT. The authors concluded that these changes might be related to increased tumor invasiveness and suggest an important role of TGFβ as inductor of EMT and metastasis [5,7]. Other studies of Brace et al. have shown that *TGFβ1*, but not *TGFβ2*, is significantly increased in PTC compared to benign thyroid nodules and therefore may serve as a potential diagnostic marker [30].

However, in our studies, no statistically significant differences were found in *TGFβ1* gene expression in BRAF V600E (+) and BRAF V600E (−) PTC groups, as well as in PTC metastatic samples with known BRAFV600E mutation status. Possibly, this may result from the fact that TGFβ has been found overexpressed locally in the invasive front of cancer cells, also in different cancer types, for example in the breast cancer [31]. Moreover, in ovarian cancer Wang et al. have demonstrated that the expression of TGF-β1 is not associated with lymphatic metastasis [32]. Moreover, it has been discovered that BRAF^V600E^ increases exclusively *TGFβ1* secretion through the MEK/ERK pathway in thyroid tumour cells [27]. The observations of Riesco-Eizaguirre et al. have led to the concept that the action of TGFβ in PTC is very important locally as a modulator of the tumor microenvironment, however, the total *TGFβ1* expression may not be altered [33].

It has been widely described that the TGFβ cascade also induces the expression of Snail-1-a zinc finger transcription factor, the main E-cadherin repressor and one of the most potent EMT inducers [34]. Snail-1 was found to be overexpressed in the invasive fronts of several human tumors derived from epithelial cells [4,14] and proved to play a key role in PTC, as well. Although Snail-1 is not expressed in normal thyroid tissue, histopathological analyses have proved that the expression of Snail-1 protein was mainly located in the cytoplasm and nuclei of PTC cells [19]. The overexpression of this gene and the upregulated protein level have been discovered in numerous cancer types such as ovarian and breast carcinomas, melanomas, oral squamous carcinomas, human thyroid carcinomas and their metastases. In the context of thyroid cancers, the effects of Snail-1 interactions have not been thoroughly studied or understood. It has been suggested that Snail-1 expression, together with TGFβ1, is associated with lymph node metastasis in PTC and may be a potential biomarker of tumor diagnosis and prognosis in PTC [8].

Interestingly, Baquero et al. have discovered that Snail-1 expression may also be regulated by BRAF^V600E^—the most common mutation found in PTCs [25]. In the thyroid carcinoma cell model, BRAF^V600E^ induced EMT via changes in Snail-1 and E-cadherin protein expression levels, which in turn increased the migration and invasiveness of these cells. Moreover, inhibition of BRAF^V600E^ significantly decreased invasion of thyroid cancer cells, tumor volume and metastases in a mouse model of anaplastic thyroid carcinoma [25,27].

In our studies, it was observed that BRAF^V600E^ mutation was present in significantly more cases of non-metastatic aggressive tumors (with T3–T4 classification). However, due to the limited number of patients in the studied groups, such statistics should be evaluated with some perspective.

Most clinical research has demonstrated an association between BRAF^V600E^ mutation status, aggressive clinicopathologic characteristics of PTC and high tumor recurrence, although the results remain controversial [24]. Contradictory results from other groups have stressed that, although BRAF^V600E^ mutation may show very high prevalence of the *BRAF* mutation in PTC in the population, the presence of the mutation itself was not statistically associated with a metastasis, patient age, completeness of resection, local invasion or tumor size score. Although BRAF^V600E^ analysis may have some value in distinguishing malignant and benign thyroid tumors, it is currently believed that alone it is not a good predictor of more the aggressive clinical course of PTC [28].

Therefore, we have aimed to evaluate if *Snail-1* and *TGFβ1* genes expression correlate with one another in human PTC tissue samples or whether they are related to BRAF^V600E^ mutation status or LNM tumor staging. We demonstrate that *Snail-1* gene expression is significantly increased in metastatic PTC patients group (based on LNM scale) in comparison to non-metastatic samples. Moreover, we show that *Snail-1* elevated expression also correlates with BRAF^V600E^ positive status. Our results clearly correspond with the data obtained by Baquero et al. on the cellular model [25]. On the contrary, Mitchell et al., utilizing immunohistochemical techniques, suggested that Snail-1 protein expression did not correlate with BRAF status and E-cadherin expression in PTC [35]. Such discrepancies may result from different molecular techniques, or the relatively small number of studied patient samples. It must be also emphasized that Snail-1 protein is relatively unstable, and in the cytoplasm, it may be prone to proteasomal degradation, with a half-life of only twenty-five minutes. GSK3β is a Snail kinase can bind to and phosphorylate this transcription factor, facilitating its degradation. Phosphorylation determines Snail-1′s subcellular location, as GSK-3β-mediated phosphorylation induces Snail-1 export to the cytoplasm [9,36]. Therefore, every study concerning Snail-1 protein expression should also consider its localization.

Interestingly, for the first time using human PTC tissue model, our findings reveal that *Snail-1* gene expression was significantly higher exclusively in metastatic PTC that harbored the BRAF^V600E^ mutation, but not in metastatic BRAF^V600E^-negative PTC tissue. In contrast, we did not observe such differences in *Snail-1* gene expression in non-metastatic PTC patients, regardless of BRAF^V600E^ mutation status. This may suggest that in cells altered by metastatic processes, such as EMT, BRAFV^600E^ mutation facilitates the mechanism of *Snail-1* overexpression. It has previously been proposed that BRAF^V600E^ can increase Snail-1 expression both at transcriptional level and via GSK3β inhibition [27]. However, the mechanism of transcription regulation in the presence of BRAF mutation remains to be further elucidated.

Despite some limitations of our study resulting from the relatively small sample size due to the selection of metastasizing PTC cases, caused by only one mutation of individual gene (BRAF^V600E^), the presented data confirm that BRAF^V600E^ participates in mechanisms involved in the increase in *Snail-1* expression in metastatic PTC. Possibly, the interplay between BRAF^V600E^ and *Snail-1* may play a key role in the thyroid gland neoplastic transformation and cancer cell invasiveness. The further elucidation of the mutual interactions between *Snail-1* and BRAF^V600E^ mutation in PTC in vivo may shed new light on the issue of metastatic thyroid cancer development. Better understanding of its complexity would help to identify specific targets for modern molecular therapies and develop more accurate diagnostic strategies.

## Figures and Tables

**Table 1:** Characteristics of papillary thyroid cancer patients from whom PTC tissue has been collected.

Characteristics	*n*	%
Sex		
Male	10	16.4
Female	51	83.6
Age (years)		
>55	13	39.3
≤55	48	60.7
T classification		
T1–T2	26	42.6
T3–T4	35	57.4
Lymph Node Metastasis		
positive	18	29.5
negative	43	70.5
Distant Metastasis		
positive	3	4.9
negative	58	95.1
BRAF^V600E^ mutation		
positive	30	49
negative	31	51

**Table 2:** Clinical features of metastatic and non-metastatic PTC tumors with or without BRAF^V600E^ mutation, ** *p* < 0.001.

Clinical Features	Metastatic Tumors			Non-Metastatic Tumors		
	BRAF^V600E^ Positive (*n* = 10)	BRAF^V600E^ Negative (*n* = 8)	*p*-Value	BRAF^V600E^ Positive (*n* = 20)	BRAF^V600E^ Negative (*n* = 23)	*p*-Value
**Gender**						
Male	4/10 (40%)	1/8 (12.5%)	0.120	2/20 (10%)	3/23 (13%)	0.400
Female	6/10 (60%)	7/8 (87.5%)		18/20 (90%)	20/23 (87%)	
**Age at Diagnosis** **(years)**						
Mean ±SD	54 ± 14	49 ± 15	0.506	55 ± 12	47 ± 15	0.050
≤55	6/10 (60%)	5/8 (62.5%)	0.501	10/20(50%)	17/23 (74%)	0.110
>55	4/10 (40%)	3/8 (37.5%)		10/20 (50%)	6/23 (26%)	
**T Classification**						
T1–T2	2/10 (20%)	3/8 (37.5%)	0.200	7/20 (35%)	18/23 (78%)	0.0002 **
T3–T4	8/10 (80%)	5/8 (62.5%)		13/20 (65%)	5/23 (22%)	

## Abbreviations

PTC	Papillary thyroid carcinoma
AT	Anaplastic thyroid carcinoma
EMT	Epithelial to mesenchymal transition
TGF	Transformation growth factor
CSC	Cancer stem cells
LNM	Lymph node metastases
MAPK	Mitogen-activated protein kinase
TUBB3	Tubulin –β3, RTK-Receptor tyrosine kinases
